# Influence of skin incision position on physiological and biochemical changes in tissue after primary total knee replacement – A prospective randomised controlled trial

**DOI:** 10.1186/s12893-015-0021-5

**Published:** 2015-04-16

**Authors:** David Q Donaldson, Matthew Torkington, Iain C Anthony, Eugene F Wheelwright, Mark JG Blyth, Bryn G Jones

**Affiliations:** Orthopaedic Research Unit, Glasgow Royal Infirmary, Gatehouse Building, 84 Castle St, Glasgow, , G4 OSF UK

## Abstract

**Background:**

Influence of skin incision position on physiological and biochemical changes in tissue after primary total knee replacement. A prospective randomised controlled trial.

The blood supply to the skin covering the anterior knee has been shown to arise predominantly from blood vessels on the medial side of the knee. Skin incisions for primary Total Knee Replacement (TKR) positioned medially therefore risk creating a large lateral skin flap that may be poorly perfused. Poorly perfused skin is likely to result in hypoxia at the wound edges and consequently may lead to delayed wound healing and complications.

**Methods:**

We have carried out a randomised controlled trial (n = 20) to compare blood flow on both the medial and lateral sides of two commonly used skin incisions in TKR (midline and paramedian). We have also assessed interstitial biochemistry (glucose, pyruvate and lactate levels) in the presumed at risk lateral skin flap of both incision types.

**Results:**

In both incision types tissue hyper-perfusion occurs post-operatively and is maintained for at least 3 days. We found no significant difference between blood flow between the two incision types on the medial side of the incision at either day 1 (p = 0.885) or day 3 post-op (p = 0.269), or, on the lateral side of the incision (p = 0.885 at day 1, p = 0.532 at day 3). Glucose levels are maintained post-operatively in the at risk lateral flap with only minimal changes. Lactate levels rise post-operatively and remain elevated for at least 24 hours. However, the levels did not reach levels suggestive of critical ischaemia in either incision group and no significant difference was observed between incision types.

**Conclusion:**

We conclude that the use of a paramedian incision results in only minimal biochemical changes, which are unlikely to alter wound healing.

**Trial registrations:**

ISRCTN06592799.

## Background

Wound healing complications are one of the most common problems encountered after Total Knee Replacement (TKR) and utilises significant additional healthcare resources including additional Health Care Worker visits for wound care, additional General Practitioner (GP) visits and occasionally re-admission to hospital. Patients who develop early wound problems are at a significantly higher risk of developing deep infection and consequently undergoing revision surgery than patients who do not have wound complications [1]. A number of risk factors are associated with development of wound complications following TKR. Patient specific factors include co-morbidities such as prior immunosuppression, including immunosuppressive therapy, hypokalaemia, malnutrition, diverticulosis, infection, diabetes mellitus, obesity, smoking, renal failure, hypothyroidism and alcohol abuse [2-4]. A number of surgical factors may also alter the risk of post-operative wound complications; including venous thromboembolic (VTE) prophylaxis, use of surgical drains and historically type of incision utilised.

**Figure 1:**
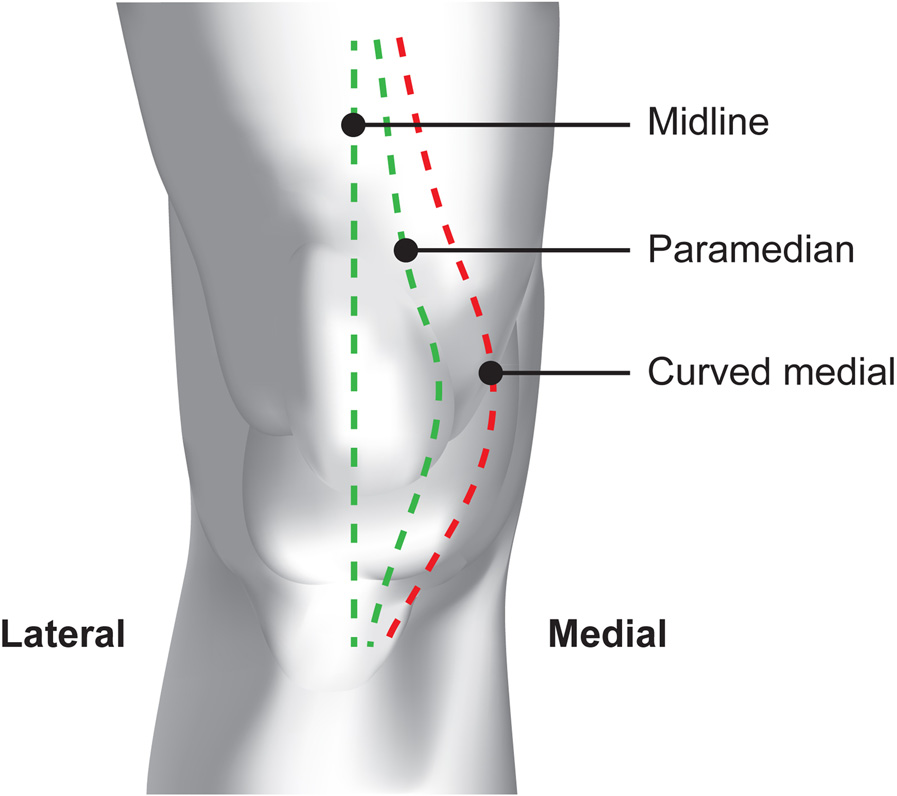
Knee Incisions.

Various surgical approaches to the knee have been described [5], the most common utilising either the midline or paramedian skin incisions – Figure 1.

The blood supply to the anterior knee arises predominantly from the medial side from anastomoses of the femoral and popliteal arteries [6]. This medially based blood supply implies that more medial based skin incisions, such as the paramedian incision, will interrupt the blood supply close to its source and theoretically leave a lateral skin flap that is poorly perfused. The midline incision however involves elevation of fascial layers with potential undermining of the skin and potential disruption of the vascular supply to the wound edges [6]. Wound breakdown can lead to direct exposure of the deep arthrotomy wound in the case of the paramedian incision and indirect exposure in the case of the midline incision. There is little evidence in the literature to support the notion that poorly perfused lateral skin flaps lead to wound complications. Furthermore, to date there have been no studies examining directly the relationship of blood flow or tissue interstitial biochemistry in relation to knee incision choice.

Johnson et al. evaluated a small cohort of 16 knees randomised to 3 different skin incisions (midline, paramedian and curved medial) and found that although the lateral skin flap always had lower post-operative oxygen tension than the medial flap, there was no significant difference between the 3 incision types [7]. Having an average of 5 knees per incision type, the study was relatively under-powered but cautioned against the use of the curved medial approach.

We have undertaken a randomised controlled trial in order to determine the influence of skin incision position on blood flow to the skin covering the anterior knee and also, we believe, the first study of its kind to evaluate tissue biochemistry at the incision site. We hypothesise that the lateral flap of the skin incision used for routine arthroplasty may be at risk from the effects of hypoperfusion.

## Methods

**Figure 2:**
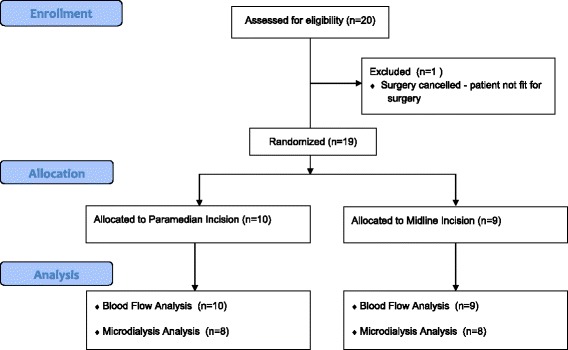
CONSORT Diagram.

A prospective controlled randomised study of 20 patients undergoing primary TKR was carried out between June 2011 and May 2012. 1 patient was withdrawn from the study prior to receiving surgery as the patient had become medically unfit for surgery (Figure 2 –CONSORT diagram). Patient demographics are detailed in Table 1.

**Table 1:** **Patient pre-operative demographics**

**Demographic**	**Midline**	**Paramedian**	**P value**
Mean age (years)	68	74	0.133
Sex (% female)	67%	70%	1.0
BMI	32	31	0.706
Diagnosis – osteoarthritis of the knee	100%	100%	1.0

Ethical approval for the study was granted by the West of Scotland Research Ethics Committee. Inclusion criteria included patients over the age of 18 with degenerative osteoarthritis of the knee and the exclusion criteria were patients with significant cardiovascular/peripheral vascular disease or a BMI of over 35. Informed consent was obtained from all patients prior to enrolment in the study. An online web based randomisation programme provided by sealedenvelopes.com with Random Permuted Blocks was used to randomise patients to incision type using a 1:1 ratio. Randomisation was carried out at the time of recruitment by the researcher taking consent. Prior to surgery, the proposed incision was marked on the patient’s knee in indelible ink. Patients were randomly allocated to midline or paramedian incisions, to create a fasciocutaneous skin flap see Figure 1. A wide curved medial incision was not used in this study.

All patients received a posterior cruciate substituting fixed bearing cemented NextGen TKR (Zimmer). Implantation was under the direct supervision of one of three orthopaedic consultants (MB, BJ, EFW) at Glasgow Royal Infirmary in a dedicated orthopaedic theatre with laminar airflow. Minimally invasive surgical techniques were not used. All patients received antibiotic prophylaxis of intravenous Cefuroxime 1.5 g prior to inflation of a tourniquet at 300 mmHg. Wounds were closed in a standard fashion with an absorbable subcuticular suture used for skin closure. No drains were employed. All patients were mobilised fully weight bearing with unrestricted active flexion and extension allowed post operatively.

As the skin incision was visible after surgery it was not possible to blind the assessors to the randomisation outcome.

**Figure 3:**
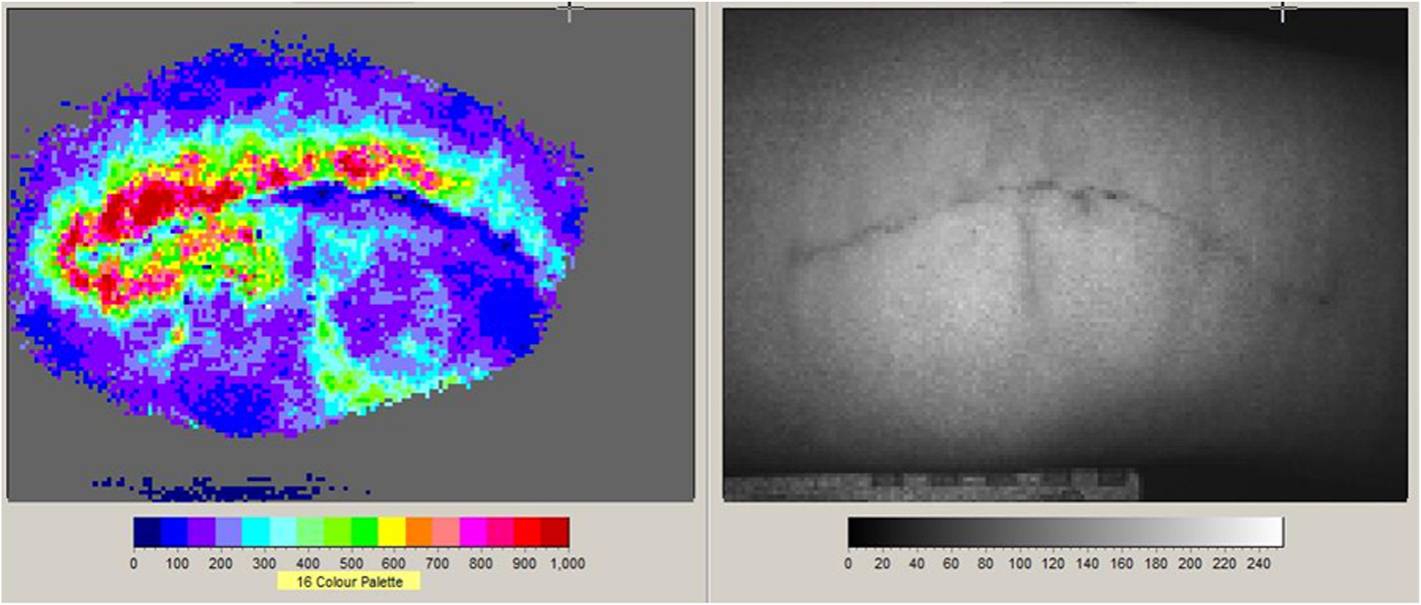
Blood Flow Imaging.

The primary outcome measure was cutaneous blood flow and was measured using Laser Speckle Contrast Blood Perfusion Imager, Moor FLPI (Moor Instruments, Axminster, UK). Images of each knee were captured pre-operatively on the ward, either the day before surgery or the morning of surgery. Post-operatively images were captured 1 day after surgery (Figure 3) and again 3 days after surgery. The image capture technique was standardised for ambient light exposure and a distance of 30 cm between camera and subject was used throughout. A reference marker for size was included in the periphery of each field to allow standardisation of images during analysis. Each recording consisted of static pictures taken every second for 10 frames. Moor FLPI V3.0 PC Software was used for image analysis and this yielded a semi-quantitative colour pixelated image. These images were processed and quantified by setting 12 Regions of Interest (ROI) along the wound in a pre-defined pattern, each region consisted of a 1 cm sq boxed area.

Secondary outcome measures included change in interstitial fluid biochemistry (Glucose, lactate and pyruvate). Interstitial fluid samples were taken from the lateral skin edge of the proposed incision pre-operatively and of the wound at 0.5, 1, 2, 4, 6, 12 and 24 hourly intervals thereafter. The samples were obtained using an indwelling subcutaneous catheter with microdialysis membrane and CMA 70 pump with a perfusate of Ringers saline suspension with infusion rate of 0.2 μL/min. The resultant dialysate vials were collected after 30 minutes infusion time, stored in airtight containers and chilled at 2 degrees centigrade, before being processed in batches. Logistical and ethical constraints restricted the use of microdialysis catheters to just one site per patient. As the lateral skin flap was thought to be at greatest risk of hypoxic damage we opted to only sample from the lateral flap of each incision. A CMA 600 microdialysis analyser on-site (CMA, Prospect Diagnostics Limited, Derbyside, UK) was used to measure the concentrations of glucose, lactate, pyruvate and hence the lactate/pyruvate ratio. The transfer of metabolites from capillary to tissue is known to be flow-limited at resting blood flows and therefore small variations in the relationship between blood flow and glucose consumption will lead to corresponding variations in the interstitial glucose concentration [8]. A large change in lactate/pyruvate ratio indicates limitation of oxygen delivery to tissues. High levels of lactate indicates tissue hypoxia [9].

Three catheter corruptions occurred during catheter collection and therefore the number of patients with a full data set for tissue biochemistry was limited to 16.

Skin sensation around the knee was assessed pre-operatively and at 3 months post-operatively using cotton wool and a blunt pin. Areas of sensory deficient were recorded on a diagram of the knee.

Comparisons between blood flow levels for both incision types were made using a Mann–Whitney test. Two way repeated measures ANOVA was used to compare biochemical markers at multiple time points.

This trial is registered with International Standard Randomised Controlled Trial Number Register - ISRCTN06592799.

This trial was originally developed as a pilot study as no relevant data was available on which to base a power calculation and therefore no formal sample size calculation was performed.

## Results

Cutaneous blood flow increased on both the medial and lateral sides of both incisions over the 3 days that data was recorded – Tables 2 and 3. This increase in blood flow was observed equally between midline and paramedian incisions. In both incision types tissue hyper-perfusion occurs post-operatively and is maintained for at least 3 days. We found no significant difference between blood flow between the two incision types on the medial side of the incision at either day 1 p = 0.885 or day 3 post-op p = 0.269 (Table 2), or, on the lateral side of the incision p = 0.885 at day 1 and p = 0.532 at day 3 post-op (Table 3). When medial and lateral flaps were compared within each incision type no significant difference was found between the blood flow on the medial side and the lateral side of the incision for either incision type, although there was a trend for the medial flap blood flow to be slightly greater.

**Table 2:** **Medial flap blood flow**

	**Incision position**		
**Time point**	**Midline**	**Paramedian**	**P value**
Pre-op median (Q1,Q3)	124 (88–152)	85 (69–112)	0.198
Day 1 median (Q1,Q3)	241 (220–270)	185 (151–357)	0.885
Day 3 median (Q1,Q3)	262 (203–326)	416 (284–439)	0.269

**Table 3:** **Lateral flap blood flow**

	**Incision position**		
**Time point**	**Midline**	**Paramedian**	**P value**
Pre-op median (Q1,Q3)	95 (84–122)	79 (63–131)	0.351
Day 1 median (Q1,Q3)	193 (164–227)	212 (155–260)	0.885
Day 3 median (Q1,Q3)	241 (171–387)	319 (195–381)	0.532

**Figure 4:**
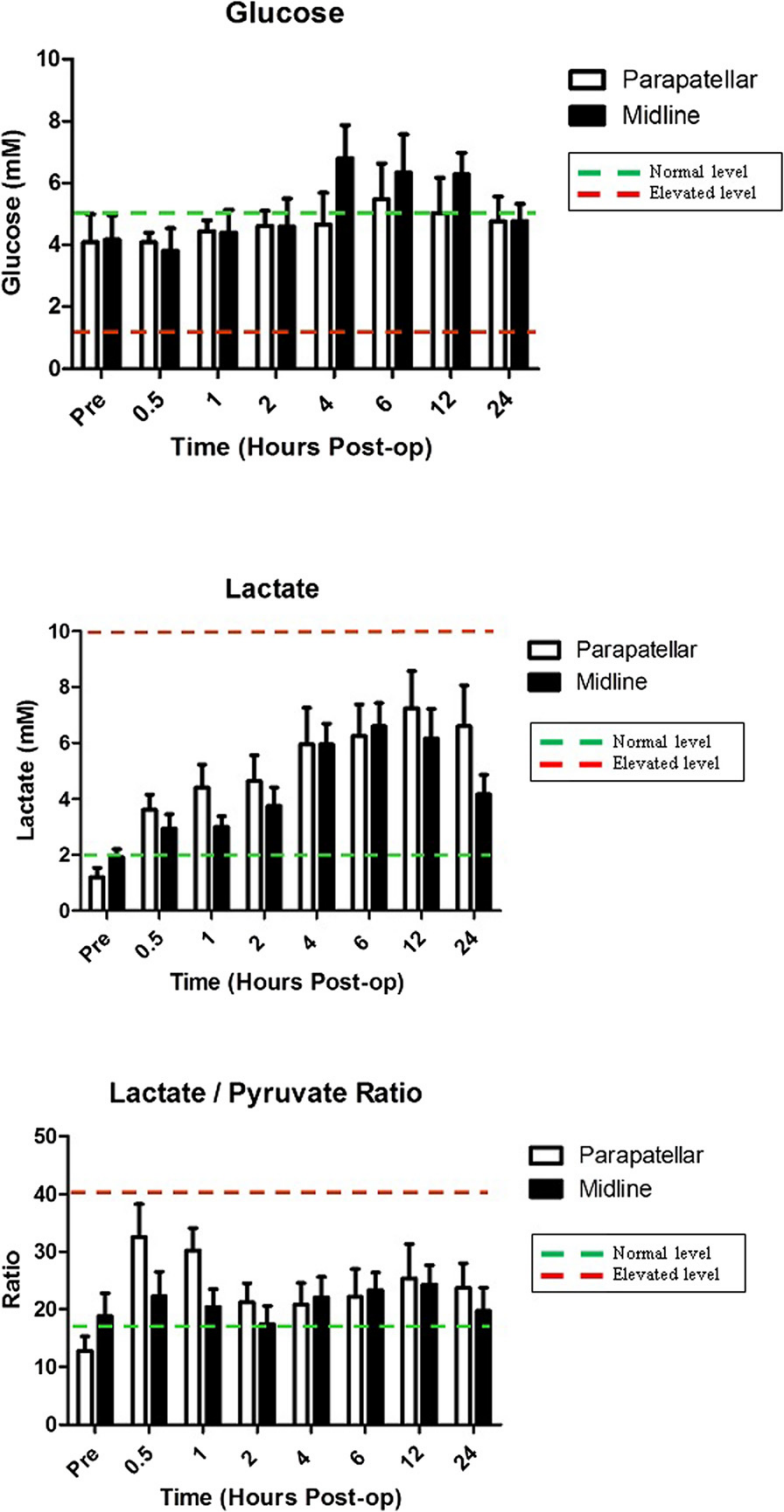
Glucose, Lactate and Lactate/Pyruvate Ratio.

The concentration of interstitial glucose remained constant and at physiological levels on both sides of the incision for both incision types throughout the monitoring period (Figure 4). No decrease in glucose was observed at any time point.

In contrast lactate levels increased immediately post-operatively and continued to rise until 6–12 hours post-operatively. At 24 hours post-operatively lactate levels in both incision groups had started to fall back towards normal, but neither group had reached normal lactate levels by the end of the monitoring period. However peak lactate levels remained well within physiological levels in both groups throughout the monitoring period (Figure 4).

The lactate/pyruvate ratio increased slightly post-operatively in both incision groups and remained mildly elevated throughout the monitoring period, but within normal physiological parameters and no significant differences were noted between the two incision types with regard to lateral skin flap lactate/pyruvate ratio (Figure 4).

Finally no difference was noted between incision types with regard to skin sensation over the knee. No patient had a sensation deficit prior to surgery, but all except one patient developed a sensation deficit over the lateral skin flap post-operatively. Although the area of sensation loss could be quantified, differences in patient anatomy and habitus made direct comparison between the two groups difficult.

No adverse events related to the study interventions were recorded in either group.

## Discussion

Several approaches to the knee have been described for Total Knee Replacement and these have been further refined to allow different arthrotomy approaches and minimally invasive techniques. However, the standard midline and paramedian incisions with parapatellar arthrotomy are probably the most commonly used incisions. Despite this there is very little evidence in the literature to distinguish the advantage of one incision over another. Uncomplicated wound healing is essential to provide a protective soft tissue envelope for a successful TKR [10]. Fortunately wound complications in primary TKR are relatively rare with early return to theatre for wound problems having an incidence of only 0.33%. However this re-intervention is associated with a significantly increased risk of deep infection (6.0%) and requirement for further surgery (5.3%) at 2 years compared with rates of 0.8% and 0.6% for patients with uncomplicated wound healing [1].

A number of factors can contribute to poor surgical wound healing, but most are outwith the direct control of the surgeon except the choice of skin incision and soft tissue handling . A midline incision for primary TKR is often favoured as it results in generation of a smaller lateral based flap. The blood supply to the skin covering the anterior knee has been shown to have a more dominant supply from the medial side [6] and any incision over the knee therefore theoretically risks leaving a poorly perfused lateral flap that must rely on the smaller supply from the lateral geniculate vessels for tissue oxygenation. The paramedian incision creates a larger lateral based flap than the midline incision, potentially further increasing the risks associated with poor oxygenation. However, the paramedian incision adheres better to Langer’s lines resulting in decreased physical tension across the wound during knee flexion [11] which is likely to aid wound healing.

This study used modern laser techniques to compare cutaneous blood flow on both sides of the surgical incision. The normal tissue reaction to trauma is one of inflammation that results in hyperaemia and swelling. Clinically this has long been observed as an increase in local temperature and erythema. The increased blood flow is facilitated by blood vessel dilatation and shunting via vessel anastomoses. It is therefore not surprising that our results confirmed an increase in overall blood flow to the skin surrounding the knee incision post surgery. We have shown that under normal circumstances physiological changes, post surgery, allow maintenance of blood flow to either side of the wound irrespective of incision choice. Our results are similar to those of Aso et al. who found no difference in blood flow between the medial and lateral side of a midline incision [12].

Previous studies assessing the effect of wound incisions on the blood supply to the skin have used skin oxygen tension as a surrogate marker of tissue perfusion and have varied in the reporting of areas of poor wound perfusion around the knee [7]. These variable results may have been secondary to tissue oedema and its influence on the ability to accurately measure capillary oxygen tension.

We believe this study is the first to directly assess the effects of incision placement on tissue perfusion at a cellular level. There is a paucity of data/studies describing levels of biochemical markers consistent with critical levels of tissue ischaemia, however we believe these better describe changes at cellular level than previous studies using oxygen tension or blood flow as a surrogate marker for tissue perfusion.

Our data suggest that neither the lateral skin flap of a midline nor paramedian incision are at significant risk of hypo-perfusion and consequently poor oxygenation. On the contrary, hyper-perfusion occurs on both sides of the incision with only minimal transitory differences in cellular biochemistry between the medial and lateral flaps, at least over the first 3 post-operative days and normal physiological glucose levels are maintained. Although interstitial lactate levels rise post-operatively these do not appear to routinely reach critical levels in the at risk lateral skin flap. Our study was unable to determine if the increased lactate levels are due to increased local production of lactate or diffusion from surrounding tissues as a result of surgical trauma to nearby soft tissue and bone or indeed from a period of tourniquet induced ischaemia. It is clear from our data, however, that regardless of source of increased lactate, wound incision choice does not have a significant effect on skin flap ischaemia as measured by cellular biochemistry.

This study was designed as a pilot study to determine sample size for a larger study. Our hypothesis was that there would be hypoperfusion of the lateral skin flap after surgery. However, we have found no evidence to support this hypothesis and therefore find no justification to undertake a larger study.

Limitations of this study include small sample size. Furthermore whilst we were able to compare blood flow on both sides of the wound we only sampled tissue metabolites from the lateral side of the wounds. Therefore, despite the theory that the blood supply around the anterior knee is more medially based and less likely to be critically lowered, we are unable to comment on the tissue metabolism of the medial skin flap. Moreover, our biochemical data only relates to the middle of the midline incision or apex of the paramedian incision.

Despite these limitations, we believe this is an important study presenting new data that aids our understanding post surgical wound blood flow and changes at a cellular level and how this relates to incision choice in primary total knee replacement. On the basis of this data we believe that a paramedian incision presents no greater risk to wound healing than the traditional midline incision used for total knee replacement surgery.

## Conclusion

We conclude that the use of a paramedian incision results in only minimal biochemical changes, which are unlikely to alter wound healing.

## Abbreviations

TKR: Total knee replacement
GP: General practitioner
VTE: Venous thrombo-embolic
ROI: Region of interest
